# *In Utero* Exposure to Maternal Diabetes Is Associated With Early Abnormal Vascular Structure in Offspring

**DOI:** 10.3389/fphys.2018.00350

**Published:** 2018-04-04

**Authors:** Abdallah Dib, Cyrielle Payen, Jennifer Bourreau, Mathilde Munier, Linda Grimaud, Ziad Fajloun, Laurent Loufrani, Daniel Henrion, Céline Fassot

**Affiliations:** ^1^UMR Centre National de la Recherche Scientifique 6015, INSERM U1083, MITOVASC, University of Angers, Angers, France; ^2^University Hospital of Angers, Angers, France; ^3^Reference Center for Rare Disease of Thyroid and Hormone Receptors, University Hospital Angers, Angers, France; ^4^Faculty of Sciences III, Azm Center for Research in Biotechnology and Its Applications, Doctoral School of Science and Technology, Lebanese University, Tripoli, Lebanon

**Keywords:** maternal diabetes, fetal programming, vascular structure, remodeling, blood flow, blood pressure

## Abstract

**Aim/hypothesis:** *In utero* exposure to maternal diabetes increases the risk of developing hypertension and cardiovascular disorders during adulthood. We have previously shown that this is associated with changes in vascular tone in favor of a vasoconstrictor profile, which is involved in the development of hypertension. This excessive constrictor tone has also a strong impact on vascular structure. Our objective was to study the impact of *in utero* exposure to maternal diabetes on vascular structure and remodeling induced by chronic changes in hemodynamic parameters.

**Methods and Results:** We used an animal model of rats exposed *in utero* to maternal hyperglycemia (DMO), which developed hypertension at 6 months of age. At a pre-hypertensive stage (3 months of age), we observed deep structural modifications of the vascular wall without any hemodynamic perturbations. Indeed, in basal conditions, resistance arteries of DMO rats are smaller than those of control mother offspring (CMO) rats; in addition, large arteries like thoracic aorta of DMO rats have an increase of smooth muscle cell attachments to elastic lamellae. In an isolated perfused kidney, we also observed a leftward shift of the flow/pressure relationship, suggesting a rise in renal peripheral vascular resistance in DMO compared to CMO rats. In this context, we studied vascular remodeling in response to reduced blood flow by *in vivo* mesenteric arteries ligation. In DMO rats, inward remodeling induced by a chronic reduction in blood flow (1 or 3 weeks after ligation) did not occur by contrast to CMO rats in which arterial diameter decreased from 428 ± 17 μm to 331 ± 20 μm (at 125 mmHg, *p* = 0.001). In these animals, the transglutaminase 2 (TG2) pathway, essential for inward remodeling development in case of flow perturbations, was not activated in low-flow (LF) mesenteric arteries. Finally, in old hypertensive DMO rats (18 months of age), we were not able to detect a pressure-induced remodeling in thoracic aorta.

**Conclusions:** Our results demonstrate for the first time that *in utero* exposure to maternal diabetes induces deep changes in the vascular structure. Indeed, the early narrowing of the microvasculature and the structural modifications of conductance arteries could be a pre-emptive adaptation to fetal programming of hypertension.

## Introduction

Nowadays, the global prevalence of diabetes in pregnant women continues to increase. Consequently, the relationship between *in utero* exposure to maternal diabetes and the incidence of cardiovascular diseases in offspring, and especially that of hypertension, has been the subject of clinical and experimental studies. Indeed, numerous works show that offspring of diabetic mothers have a higher systolic blood pressure (SBP) than offspring of control mothers (Bunt et al., 2005; Rocha et al., 2005; Wichi et al., 2005; Nehiri et al., 2008; Gomes and Gil, 2011; Aceti et al., 2012).

Increasing evidences propose that functional changes in the vasculature could be a major reason for the increase of blood pressure in offspring of diabetic rats (DMO) (Manderson et al., 2002; Rocha et al., 2005; Simeoni and Barker, 2009; Duong Van Huyen et al., 2010; Porto et al., 2010; Vessières et al., 2016). In experimental studies, it is well known that exposure to maternal diabetes induces vascular dysfunction in large arteries of DMO, reflected by a reduced response to endothelium-dependent vasodilators and an increased contractile response. Indeed, Duong *et al* have demonstrated a specific gene expression profile of the thoracic aorta in favor of vasoconstriction with altered prostacyclin-induced vasodilation, linked to a reduction of prostacyclin receptor expression (Duong Van Huyen et al., 2010). Endothelial dysfunction of the microcirculation has also been described; functional studies show that endothelium-mediated vasodilation induced by acetylcholine is reduced (Holemans et al., 1999; Rocha et al., 2005; Ramos-Alves et al., 2012). Recently, we have shown that in resistance arteries of old DMO rats, endothelial vasodilator dysfunction was exacerbated and pressure-induced (myogenic) tone was maintained at a high level (Vessières et al., 2016).

Because of a close relationship between vascular tone and wall structure (Bakker et al., 2002), modifications of vascular tone are able to induce structural adaptation of vessels. It is well known that persistent vasoconstriction induces inward remodeling in several types of arteries (Bakker et al., 2002, 2004, 2005). This type of remodeling could be inhibited or reversed by vasodilator compounds such as calcium channel inhibitors (Bakker et al., 2002) or transglutaminase 2 inhibitor like cystamine (Eftekhari et al., 2007). Thus, modifications of vascular tone could not only be involved in the development of hypertension but also have an impact on vascular structure.

Even if the relation between prenatal conditioning of hypertension and microvascular function is well documented in the case of *in utero* exposure to maternal diabetes, the impact on vascular structure and blood flow variations have not yet been studied. Moreover, alterations of the vascular structure could influence remodeling mechanisms and responses to hemodynamic variations. In addition maladaptive vascular remodeling is currently recognized as an important contributor to the development of cardiovascular pathologies (Pasterkamp et al., 2000). Thus, our objective was to study the impact of *in utero* exposure to maternal diabetes on vascular structure on basal conditions (3 months of age) and remodeling induced by chronic hemodynamic changes.

## Materials and methods

### Animals

Pregnant Sprague–Dawley rats, weighing 250–300 g, were made diabetic on day 0 of gestation by a single intraperitoneal injection of streptozotocin (35 mg/kg, Sigma, St Quentin Fallavier, France) as previously described (Duong Van Huyen et al., 2010). The diabetic state was checked in fasted rats by measuring the plasma glucose concentration (AccuChek®, Roche, Boulogne-Billancourt, France). Only pregnant females whose plasma glucose ranged between 300 and 450 mg/dl were included in the study. This diabetic status was confirmed every 2 days until delivery (Figure S1). On the day of delivery the newborn rats were weighed. Each litter was then reduced to 10 pups. All animals were kept in a temperature and light controlled room, at 21°C with a 12 h light cycle. They had access to food (diet n°3430, Serlab, Compiègne, France) and tap water *ad libitum*. Control animals were born of non-diabetic females (CMO). We used 30 CMO males and 30 DMO males from at least 6 different litters for each group. One animal per litter is included in each experimental group. Before sacrifice, glycemia of each fed animal was measured. All experiments were conducted in accordance with the institutional guidelines and the recommendations for the care and use of laboratory animals of the French Ministry of National Education, Research and Innovation. The protocol was approved by the Ethics Committee of “Pays de Loire” (permit n° 00960.01).

### Arterial blood pressure measurements

SBP was measured in conscious rats by tail-cuff plethysmography (BP-2000, Visitech, Apex, USA). Briefly, this technique uses transmission photoplethysmography in which variations of light transmitted through the tail is the basic signal that is analyzed to determine the blood pressure and pulse rate. For good quality, reliability and reproducibility of the measurements, rats were trained 1 week before experimentation. Then, blood pressure was recorded in quiet animals. For each animal, the mean SBP was averaged from 15 measurements recorded every day for 7 consecutive days.

### Model of flow-induced remodeling

Three-month old male rats were anesthetized (Isoflurane, 2.5%) and submitted to surgery in order to modify blood flow in the mesenteric circulation as described previously (Bouvet et al., 2007). Briefly, consecutive first-order arteries were used. Ligations (7-0 silk surgical thread) were applied to second-order branches of the first artery (low flow artery, LF). Equivalent arteries located at a distance from the ligated artery were used as control arteries (normal flow, NF) (**Figure 3A**). At the time of anesthesia and at the end of surgery, animals were treated with buprenorphine (TEMGESIC, 0.1 mg/kg, s.c.). After 7 or 21 days the rats were sacrificed by CO_2_ inhalation. The gut was excised and the mesenteric arteries were gently dissected. From each rat, LF and NF arteries were isolated and divided in several segments (from proximal to the distal part of the artery) for functional measurements, histological study and biochemical analyses, respectively.

### Pressure–diameter relationship in isolated mesenteric arteries

LF and NF arteries were cannulated at both ends and mounted in a video monitored perfusion system, as previously described (Retailleau et al., 2013). Briefly, cannulated arterial segments were placed in a 5 ml organ bath containing a Ca^2+^-free physiological salt solution with EGTA (2 mmol/L) and sodium nitroprusside (SNP, 10 μmol/L). Pressure was progressively increased (10–125 mmHg) in order to determine passive arterial diameter. Pressure and diameter measurements were collected using a data acquisition system (Biopac MP100 and Acqknowledge® software, Biopac). At the end, arterial segments were fixed with formaldehyde under a pressure of 75 mmHg in order to perform histological studies.

### Western blot analysis in mesenteric arteries

Western blot analysis of proteins of interest was performed. In brief mesenteric arteries were rapidly dissected and frozen. Then, proteins were extracted and the total protein content was determined by the Bradford technique in order to file equal amounts (15 μg) of the denatured proteins. Membranes were incubated with polyclonal antibodies directed against transglutaminase 2 (TG2, ab421, 1/1000 TBST-BSA 0.5%, Abcam) or mitochondrial Mn superoxyde dismutase (SOD2, SOD-110, 1/1000 TBST-BSA 0.5%, Enzo Life Sciences). The detection was performed by chemiluminescence emitted from luminol oxidized by peroxidase (ECL system, GE Healthcare, Velizy-Villacoublay, France). Each protein expression was compared to GAPDH (14C10, 1/1000 TBST-BSA 0.5%, Cell Signaling) and expressed as ratio between protein level and GAPDH.

### Reactive oxygen species (ROS) measurement

ROS detection was performed on 3-month old CMO and DMO rats. Transverse cross-sections 7 μm thick of mesenteric arteries were incubated with dihydroethydine (DHE), as previously described (Cousin et al., 2010). DHE, in the presence of superoxide, is briefly oxidized to fluorescent ethidium bromide. Ethidium bromide is trapped by intercalation with DNA, and the number of fluorescent nuclei indicates the relative level of superoxide production. Positive staining was visualized using confocal microscopy (Nikon Eclipse TE2000S) and MetaMorph® software (Molecular Devices, Sunnyvale, USA; Duong Van Huyen et al., 2010).

### Transglutaminase activity

Mesenteric arteries of 3-month old CMO and DMO rats were split into 4 groups and incubated at 37°C during 24 h in 100 μL buffer containing Leibovitz medium with 10% fetal bovine serum, a mix of antibiotic-antimycotic solution (1%, 15240062, Gibco, Courtaboeuf, France), Dapi (5 μg/mL, Molecular Probes, Carlsab, USA) and either Alexa Fluor594/Cadaverine (10 μmol/L, A-30678, Invitrogen, Carlsab, USA), or (Aceti et al., 2012) Alexa Fluor594/Cadaverine + SNP **(**10^−3^ mol/L), or (Bunt et al., 2005) Alexa Fluor594/Cadaverine + dithiothreitol (DTT, 2 mmol/L, 43816, Sigma, St Quentin Fallavier, France), or (Gomes and Gil, 2011) Alexa Fluor594/Cadaverine + DTT (2 mmol/L) + SNP (10^−3^ mol/L). Vessels were mounted on Mowiol and imaged on a confocal microscope (Nikon Eclipse TE2000S). Transglutaminase activity was quantified by spatial integration of AlexaFluor594 signal with ImageJ software. Data were corrected for vessel size and depicted in arbitrary units.

### Isolated perfused kidney

Three-month old male rats were operated under anesthesia (2.5% isoflurane). After laparotomy, renal arteries, the abdominal aorta, the superior mesenteric artery and the inferior vena cava were cleaned of the surrounding connective tissue. The kidney was released and the crossroads of the abdominal aorta with left renal artery was carefully dissected. A catheter (PE50/PE20, FMD) was inserted from the abdominal aorta to the renal artery (Weiss et al., 1959) and was maintained by a ligation on the abdominal aorta. The kidney, perfused continuously at 37°C by Krebs solution (according to El-Mas et al., 2003), was then removed carefully and placed in an organ bath (El-Mas et al., 2003) connected to a peristaltic pump to infuse kidney at given rates. The renal perfusion pressure was measured in response to stepwise increase in perfusion flow (4–50 ml/min). Pressure measurements were collected using a data acquisition system (Acqknowledge® software, Biopac, Paris, France).

### Histomorphometry analysis

Each segment of mesenteric or renal arteries and thoracic aorta were embedded in Tissue-Tek (Sakura) and frozen in isopentane. sections (7 μm) were stained with orcein in order to measure histomorphometric parameters (internal and external diameters and medial cross-sectional area, MCSA) after image acquisition (Nikon Eclipse E600 microscope, Sony camera) and analyzed using ImageJ software (NIH).

### Vascular smooth muscle cell attachment to elastic lamellae

Thoracic aorta of 3-month old CMO and DMO rats were fixed in 0.1 M phosphate buffer containing 2.5% of glutaraldehyde (LFG) overnight at 4°C. Samples were then post-fixed with 1% osmium tetroxide/1% potassium ferricyanide for 45 min and dehydrated in a graded series of ethanol. Samples were finally embedded into epoxy resin (Epon, LFG) and ultrathin sections (70 nm) were cut, contrasted with 3% uranyle acetate for 15 min and observed with a JEOL 1,400 transmission electron microscope (JEOL) at an accelerating voltage of 120 keV. Image acquisition was made at a magnification of X 12,000. Vascular smooth muscle cell attachments to elastic lamellae correspond to expansions of VSMCs composed by oxytalan fibers that span obliquely from the dense plaques of VSMCs to the extracellular matrix (ECM) (Dingemans et al., 2000). Then the number of anchorage sites between VSMCs and ECM was measured as previously described (Bezie et al., 1998). Results were obtained by analyzing 50 images per animal.

### Statistical analysis

Results were expressed as means ± SEM. Each “n” corresponds to the number of animals per group. Significance of the differences between NF and LF for pressure-diameter curves or between DMO and CMO for perfusion pressure-flow curves was determined by regular 2-way ANOVA followed by Bonferroni's multiple comparison test or a non-parametric Mann-Whitney test for histological analysis. Values of *p* < 0.05 were considered to be significant. All statistical analysis were performed using GraphPad Prism® software.

## Results

### Physiological vascular structure in rats exposed *in utero* to maternal diabetes

**Figure 1:**
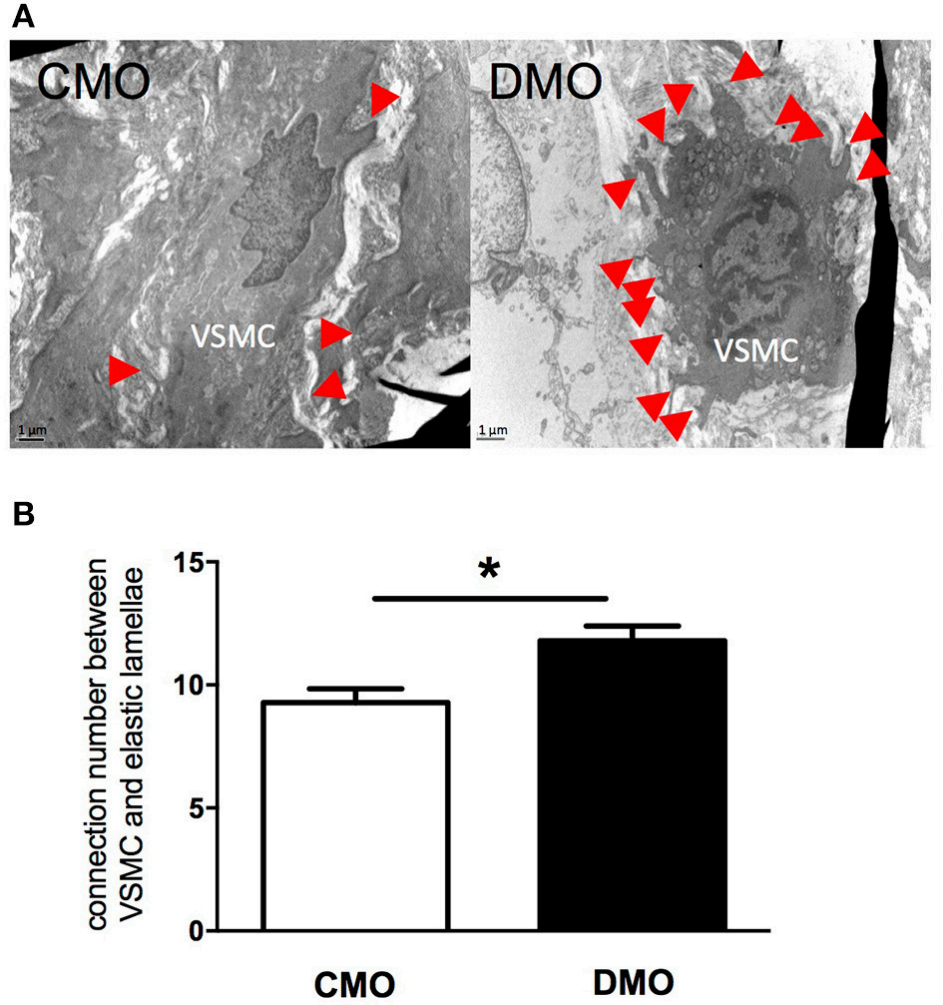
Ultrastructure analysis of thoracic aorta in 3-month old control (CMO) and diabetic (DMO) mother offspring. **(A)** Electronic microscopy images of a vascular smooth muscle cell (VSMC) and its attachments on extracellular matrix showed by red arrows; magnification X12,000. **(B)** Number of connections between VSMCs and extracellular matrix (*n* = 3 for CMO, open bars and *n* = 3 for DMO, solid bars); each bar graph represents mean ± SEM. ^*^p < 0.05 DMO vs. CMO.

At 3 months of age, DMO and CMO rats have similar physiological parameters (i.e., body weight, glycemia and mean blood pressure, MBP, Table 1). Interestingly, in basal conditions, mesenteric resistance arteries of DMO rats have a smaller diameter than those of CMO rats (Table 1). This is associated with a reduced MCSA (Table 1) indicating a basal structural reorganization of the vascular wall in these arteries. For conductance arteries, no difference in diameter and MCSA is observed between CMO and DMO rats at 3 months of age (Table 1); but we measured an increased number of connections between VSMCs and ECM in these vessels (Figure 1).

**Table 1:** Physiological parameters (body weight, glycemia and mean blood pressure, MBP) and morphology (external diameter and cross-sectional area of the media, MCSA) of resistance mesenteric artery and conductance thoracic aorta of 3-month old control (CMO) and diabetic (DMO) mother offspring.

	**CMO**	**DMO**
**PHYSIOLOGICAL PARAMETERS**		
Body weight (g)	435.2 ± 11.1 (*n* = 29)	421.8 ± 10.7 (*n* = 30)
Glycemia (mg/dl)	155.3 ± 9.4 (*n* = 29)	153.7 ± 11.4 (*n* = 30)
SBP (mmHg)	118 ± 3 (*n* = 29)	123 ± 4 (*n* = 30)
**RESISTANCE MESENTERIC ARTERY**		
External diameter (μm)	368.4 ± 9.9 (*n* = 9)	320.2 ± 12.9^**^ (*n* = 9)
MCSA (μm^2^)	15.9 × 10^3^ ± 1.6 × 10^3^ (*n* = 9)	10.3 × 10^3^ ± 0.8 × 10^3^^**^ (*n* = 9)
**CONDUCTANCE THORACIC AORTA**		
External diameter (μm)	1829.6 ± 40.1 (*n* = 12)	1894.6 ± 22.6 (*n* = 13)
MCSA (μm^2^)	414.6 × 10^3^ ± 19.5 × 10^3^ (*n* = 12)	432.7 × 10^3^ ± 19.1 × 10^3^ (*n* = 13)

** *p < 0.01 DMO vs. CMO*.

### Modification of resistance artery hemodynamic in rats exposed *in utero* to maternal diabetes

**Figure 2:**
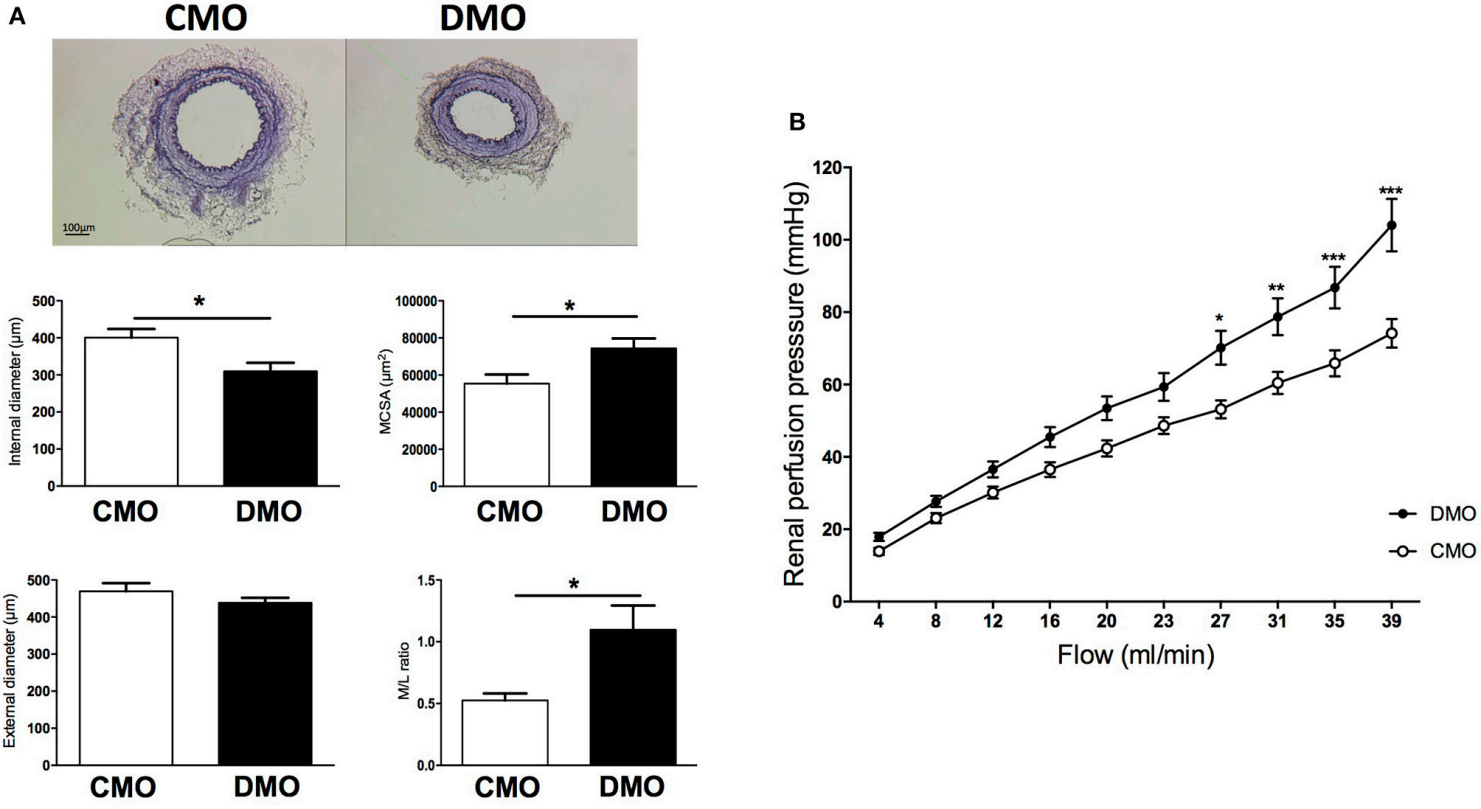
Histological and functional analysis of kidney resistance arteries in 3-month old control (CMO) and diabetic (DMO) mother offspring. **(A)** Examples of light microscopy images of renal arteries of CMO and DMO rats, magnification X4 with associated morphometric measurements of internal and external diameters, cross-sectional area of the media (MCSA) and media to lumen ratio (M/L ratio) of the renal artery in CMO (open bars, *n* = 4) and DMO (solid bars, *n* = 6) rats; each bar graph represents mean ± SEM. **(B)** Flow-response curve in isolated perfused kidney of CMO (white curve, *n* = 9) and DMO (black curve, *n* = 12) rats. ^*^p < 0.05, ^**^p < 0.01, and ^***^ *p* < 0.001 DMO vs. CMO.

Because small variations of diameter in resistance arteries could modify vascular resistances, we studied the renal arterial system, the most important microcirculatory system sensitive to flow and peripheral resistance. Under basal condition, the histological study revealed a decreased internal diameter of the renal artery associated with an increase of MCSA in 3-month old DMO compared to CMO rats, leading to an increase in media to lumen ratio in DMO rats (Figure 2A). In addition, in isolated perfused kidneys stepwise increases in flow induced a progressive rise in perfusion pressure. This flow-pressure relationship is shifted leftward in DMO compared to CMO (Figure 2B), suggesting a rise in renal peripheral vascular resistance.

### Impact of decreased flow on resistance artery remodeling

**Figure 3:**
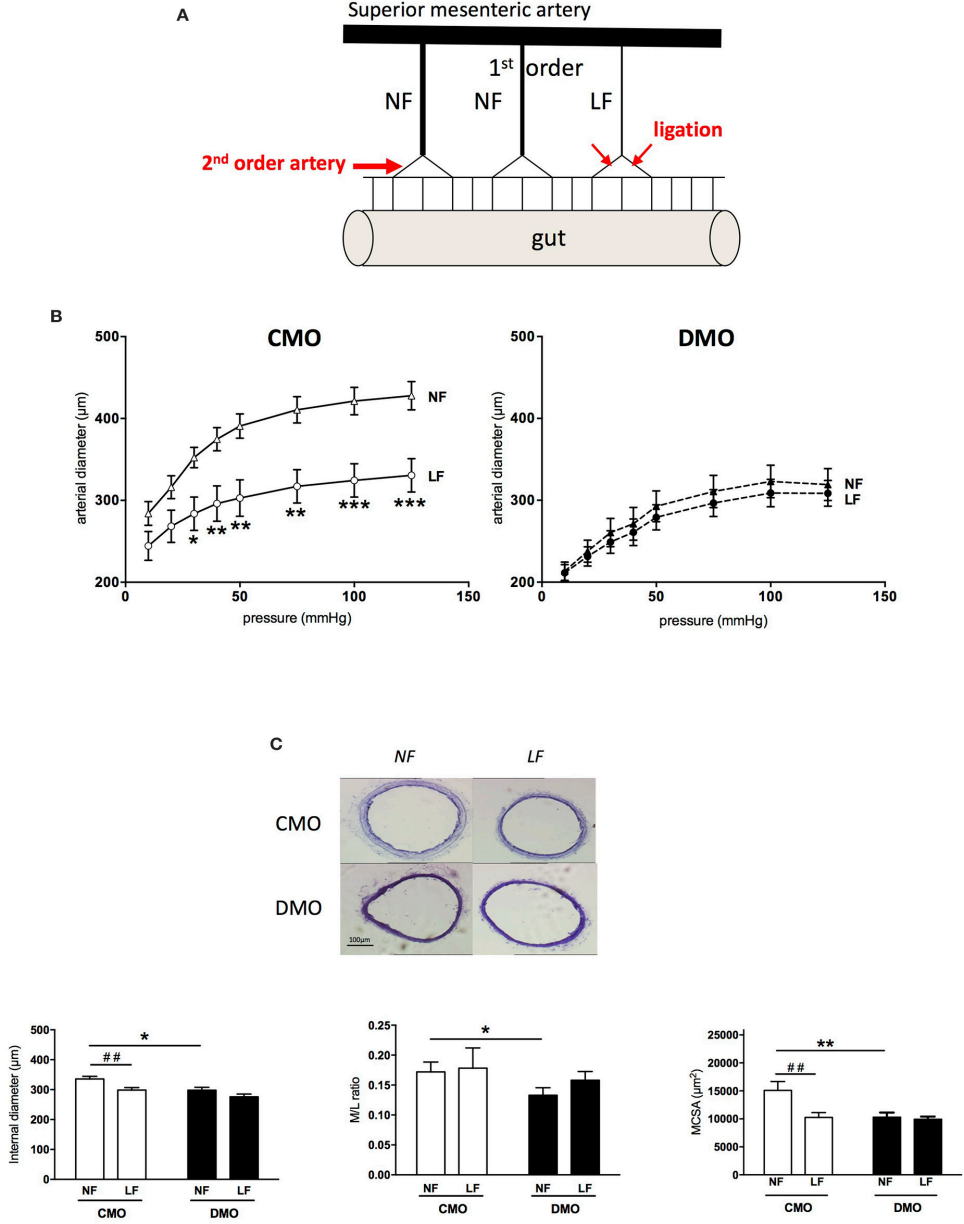
Impact of decreased-flow on mesenteric resistance arteries in 3-month old control (CMO) and diabetic (DMO) mother offspring. **(A)** Schematic representation of the model used to locally change blood flow in mesenteric arteries after ligation. **(B)** Low flow (LF) and normal flow (NF) arteries diameters of CMO and DMO 1 week post-ligation in response to stepwise increase pressure (*n* = 7), ^*^p < 0.05, ^**^p < 0.01, and ^***^p < 0.001 LF vs. NF. **(C)** Examples of light microscopy images of LF and NF mesenteric arteries of CMO and DMO rats, magnification X10 with associated morphometric measurements of internal diameter, media to lumen ratio (M/L ratio) and cross-sectional area of the media (MCSA) of normal flow (NF) and low flow (LF) arteries in CMO (open bars, *n* = 12) and DMO (solid bars, *n* = 9) rats; each bar graph represents mean ± SEM. ^*^p < 0.05, DMO vs. CMO, ^##^*p* < 0.01 LF vs. NF.

After 1 week of ligation, a stepwise increase in pressure induced a significantly increased passive diameter in NF artery than in LF artery of CMO rats (Figure 3B). This was associated with a decrease of internal diameter and MCSA (Figure 3C), with no modification of M/L ratio (Figure 3C). These results are representative of the development of an inward remodeling in LF arteries of CMO rats. Interestingly, for DMO rats, we do not observe a different behavior between NF and LF arteries in response to stepwise increase in pressure (Figure 3B). In addition, internal diameter and M/L ratio are similar between LF and NF DMO rats as well as MCSA (Figure 3C), showing an impairment of arterial response to the chronic decrease in flow. Interestingly, 3 weeks after ligation, histological parameters and passive diameter evolution of LF mesenteric artery in DMO rats were similar to those obtained 1 week post-ligation (Figure S2), which demonstrates a total absence of inward remodeling development in the case of low flow perturbations in these animals.

### Permanent oxidative stress and transglutaminase inactivity in resistance arteries of rats exposed *in utero* to maternal diabetes

**Figure 4:**
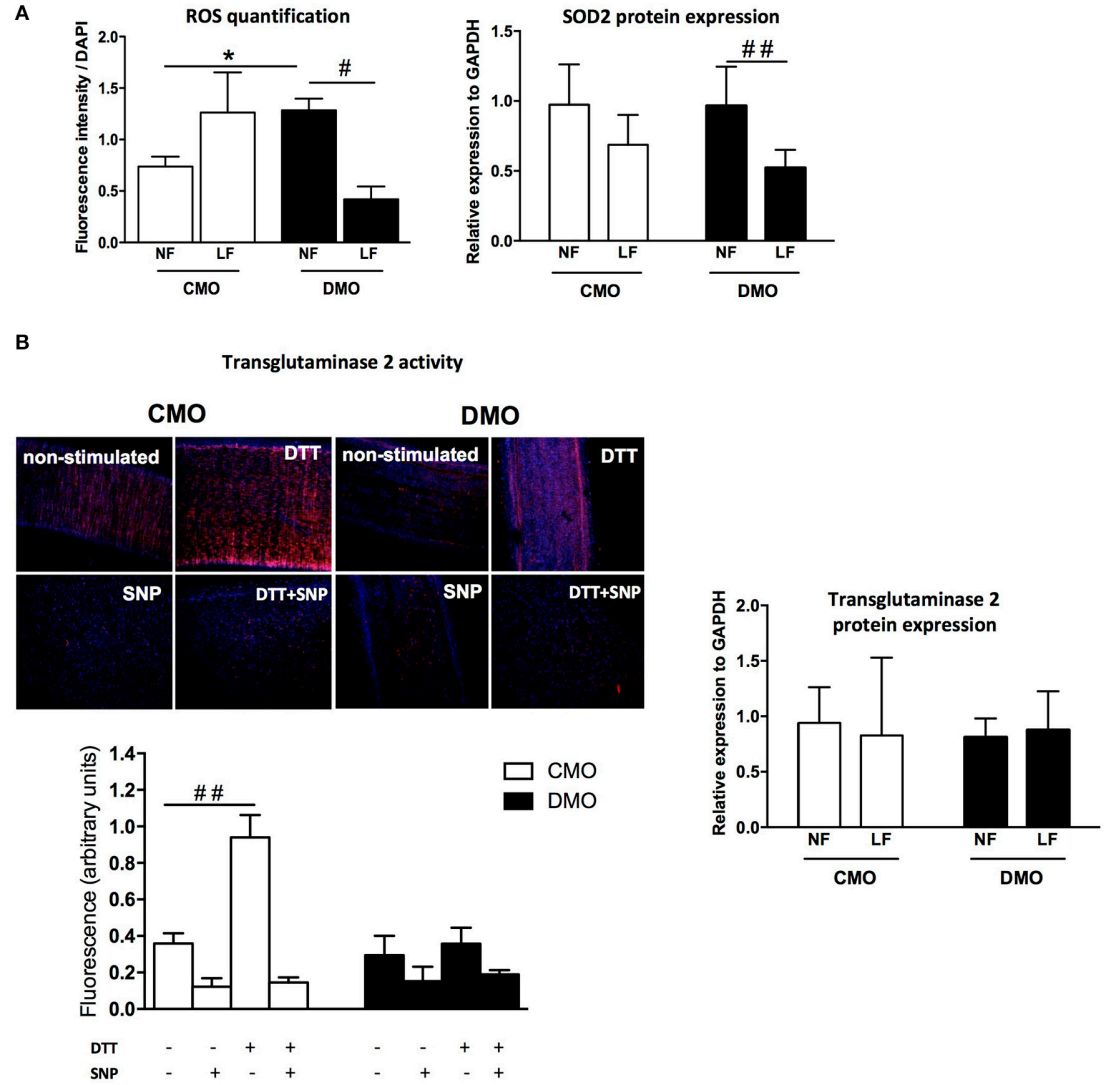
Oxidative stress and transglutaminase 2 pathways analysis. **(A)** Reactive oxygen species (ROS) measurement by detection of the number of fluorescent nuclei relative to superoxide production level (left panel) and western blot analysis of mitochondrial superoxide dismutase (SOD2) normalized to GAPDH protein expression (right panel) in normal (NF) and low (LF) flow mesenteric arteries of 3-month old control (CMO, open bars) and diabetic (DMO, solid bars) mother offspring; each bar graph represents mean ± SEM (*n* = 6 per group). ^#^*n* < 0.05 and ^##^*n* < 0.01 LF vs. NF; ^*^n < 0.05 DMO vs. CMO. **(B)** Transglutaminase 2 activity (incorporation of Alexa Fluor-594/Cadaverine) measured in non-stimulated mesenteric artery (basal condition), with SNP (NO donor, inhibitor of TG2 activity), with DTT (activator of TG2 activity) or with SNP + DTT in CMO (*n* = 6) and in DMO (*n* = 3) rats (left panel) and relative expression of Transglutaminase 2 normalized to GAPDH protein expression (western blot analysis, *n* = 6 per group) in normal (NF) and low (LF) flow mesenteric arteries of 3-month old control (CMO, open bars) and diabetic (DMO, solid bars) mother offspring; each bar graph represents mean ± SEM. ^##^*p* < 0.01 DTT vs. non-stimulated.

At a basal level, we measure a high level of ROS in NF arteries of DMO compared to CMO rats (Figure 4A), indicating an activation of the oxidative stress pathway in DMO animals. Moreover, although ROS level increases in CMO in response to decreased-flow, there is no more increase of ROS production in DMO LF arteries; on the contrary, we observe a 50% decrease of ROS level in theses LF arteries (Figure 4A). In parallel, western blot analysis demonstrates a high expression of the protective mitochondrial SOD2 in response to increased oxidative stress in DMO rats although SOD2 protein expression does not change between NF and LF in CMO rats (Figure 4A and Figure S3).

In order to test if endogenous TG2 could be activated by a cell-permeable reducing agent, mesenteric arteries were incubated with DTT. In 3-month old CMO rats, TG2 activity, as indicated by incorporation of Alexa Fluor-594/Cadaverine, is higher after stimulation by DTT than in non-stimulated vessels. Moreover, TG2 activity was strongly reduced by SNP (NO donor). Interestingly, in DMO mesenteric arteries, after DTT stimulation we detect a very low TG2 activity compared to CMO mesenteric arteries (Figure 4B). In parallel, western blot analysis does not show modification of tissue-TG2 expression level in NF and LF arteries either in CMO or in DMO (Figure 4B and Figure S3).

### Absence of inward remodeling of thoracic aorta in response to chronic high blood pressure in rats exposed *in utero* to maternal diabetes

**Figure 5:**
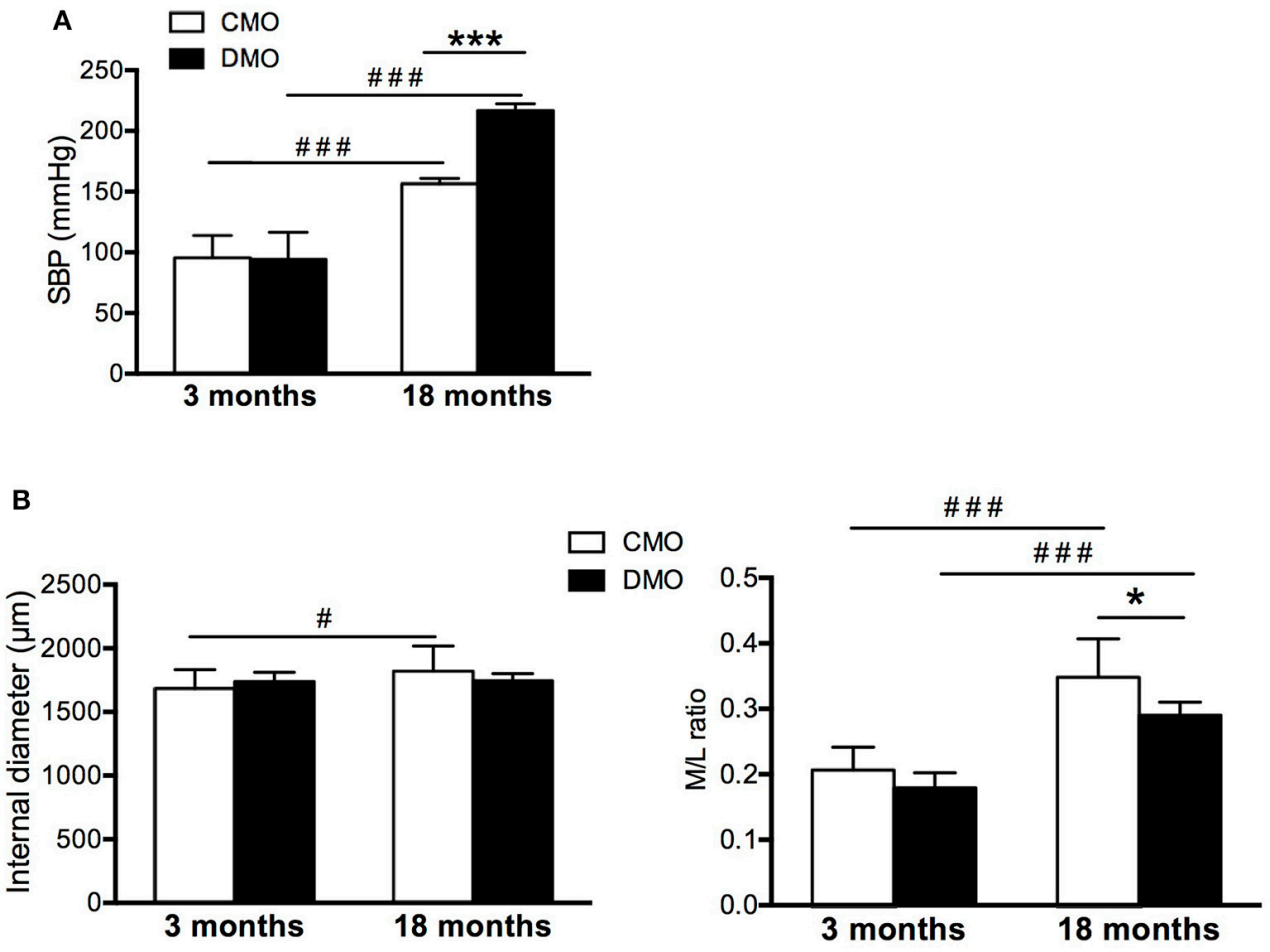
Blood pressure and morphometry of thoracic aorta at pre-hypertensive (3 months) and hypertensive (18 months) stages in control (CMO) and diabetic (DMO) mother offspring. **(A)** Evolution of systolic blood pressure (SBP). **(B)** Morphometric measurements of internal diameter and media to lumen ratio (M/L ratio) of CMO (open bars, *n* = 12 at 3 and 18 months of age) and DMO (solid bars, *n* = 13 at 3 months of age and *n* = 5 at 18 months of age) rats; each bar graph represents mean ± SEM. ^#^*p* < 0.05 and ^###^*p* < 0.001 18 months vs. 3 months of age, ^*^p < 0.05 and ^***^p < 0.001 DMO vs. CMO.

At 18 months of age, although we measure a 1.5-fold increase of SBP in CMO rats, the increase observed in DMO rats is greater (a 2-fold increase of SBP, Figure 5A). This strong increase in SBP reflects the development of hypertension in DMO animals, as previously described (Nehiri et al., 2008). Histological analysis of thoracic aorta sections did not show any difference in MCSA, external and internal diameters and M/L ratio between CMO and DMO rats at 3 months of age (Table 1 and Figure 5B). In 18 months-old animals, we measure an equivalent rise of internal diameter both in CMO and DMO despite hypertension (Figure 5B). In addition, M/L ratio is enhanced at 18 months of age (Figure 5B). Nevertheless, the magnitude of the remodeling response is less important in DMO than in CMO rats while they have the highest level of blood pressure (Figure 5A). These results show an inadaptive arterial remodeling in response to high blood pressure in DMO rats.

## Discussion

Our study demonstrates, for the first time, that *in utero* exposure to maternal diabetes induces permanent structural modifications of the vasculature. Indeed, we found deep vascular modifications of resistance (i.e., renal and mesenteric arteries) and conductance (i.e., thoracic aorta) arteries in basal conditions, but also an absence of vascular adaptation to hemodynamic perturbations (i.e., decreased flow and hypertension) in male DMO rats.

Previous studies in male DMO rats highlighted the impact of maternal diabetes on programming of hypertension; in this model arterial blood pressure rises above normal pressure values around 6 months of age (Nehiri et al., 2008). The developmental origin of hypertension during adulthood is now well admitted and linked to nutritional insults in early life both in experimental (Liu et al., 2013; Tain et al., 2017) and clinical studies (Alexander, 2006; Aceti et al., 2012; Szostak-Wegierek, 2014; Levy et al., 2017). Even if sexual dimorphism in developmental programming of hypertension is well established (Ojeda et al., 2014), a large number of studies, including ours, are particularly interested in male offspring because cardiovascular function seems to be more affected than in female offspring (Cheong et al., 2016). Indeed previous studies have highlighted a protective role of sex hormones leading to a less pronounced phenotype in female than in male offspring (Romano et al., 2015). In the present study, we demonstrate that in the absence of any hemodynamic perturbation (3 months of age), male DMO rats have already deep changes in vessel structure. First of all, electronic microscopy analysis of thoracic aorta showed an increased number of connections between VSMCs and elastic lamellae at 3 months of age. This structural modification occurred without difference in wall components (e.g., collagen and elastin levels), as shown in our previous work (Duong Van Huyen et al., 2010). This vascular wall restructuring is normally associated with sustained hypertension to produce mechanical adaptation of the arterial wall as described in spontaneously hypertensive rats (Bezie et al., 1998). In our study, despite the normal SBP observed in 3-month old DMO rats, the *in utero* exposition to maternal diabetes might induce adaptive vascular development in order to better support the programmed high blood pressure later in life.

Furthermore, in DMO rats, a narrowing of resistance arteries seems to be present in basal conditions with smaller internal and external diameters. In this study, we show that DMO rats have a high level of ROS; this result is in agreement with the high protein expression level of SOD2, an endogenous mitochondrial antioxidant, in order to counterbalance the negative effects of ROS. Interestingly, we observe the same profile of SOD2 protein expression between CMO and DMO. However, the levels of ROS in NF and LF of CMO and DMO rats are different. In fact, ROS quantification by DHE represents a global analysis of cellular and mitochondrial stress while SOD2 is bound to the protective mitochondrial oxidative stress (Sack et al., 2017). Then the difference observed between CMO and DMO reflects a different origin of ROS; in LF CMO rats, the ROS seem to originate from both cellular and mitochondrial oxidative stress although in NF DMO rats, the ROS seem to have a predominantly mitochondrial origin. Nevertheless, our observations, coupled with the fact that DMO develop a vasoconstrictor phenotype (Duong Van Huyen et al., 2010; Vessières et al., 2016), could explain the narrowing of resistance arteries observed in basal conditions. But, in the current state it is difficult to exclude an impact of *in utero* exposure to maternal diabetes on vessel development. Indeed, a recent study on chicken embryos has shown that high glucose inhibited development of the blood vessel plexus which led to the development of narrower vessels (Jin et al., 2017). On the other hand, insulin, by its positive regulation on IGF-1 (insulin-like growth factor-1) level, could influence vessel size (Piecewicz et al., 2012). Thus, a decrease in insulin content may lead to smaller vessels. Such a decrease of fasting venous blood insulin in children exposed to maternal diabetes has been recently described (Sauder et al., 2017). But in our previous work, we did not observe modification of pancreatic insulin concentration or anatomical structure of the pancreas in DMO rats (Blondeau et al., 2011).

A small decrease of a vessel diameter can dramatically increase vascular resistance (because vessel resistance is inversely proportional to the vessel radius to the fourth power according to Poiseuille's equation). Then, variations in diameter of resistance arteries can impact homeostatic systems and especially kidney hemodynamic. In this study we observe a higher-pressure response to flow in DMO isolated perfused kidney at 3 months of age, suggesting that renal vascular resistance is altered. Some studies suggest that maternal hyperglycemia can promote remarkable changes both in kidney morphology and function. Then, the increase of vascular resistance could be linked to the basal hypertrophic remodeling (increased MCSA) of renal resistance artery that we described, but also to a decrease in nephron number since others studies have shown an impaired nephrogenesis with a reduction of 30% of nephron number in association with a decreased renal function in DMO rats (Amri et al., 1999; Nehiri et al., 2008). This impaired kidney development would be the consequence of an ureteric branching morphogenesis reduction (Hokke et al., 2013). Nevertheless, modifications of kidney hemodynamics are directly implicated in hypertension. Brenner et al. have been the first to establish a relationship between intra-uterine environment, decreased nephron number and the development of hypertension (Brenner et al., 1988). Clinical results show, in association with a higher SBP, that adult offspring of type 1 diabetic mothers have a reduced renal functional reserve, which may reflect a decreased number of nephrons (Abi Khalil et al., 2010). As in the case for decreased nephron number, resistance artery remodeling could be implicated in the development of hypertension during adulthood.

Inward remodeling is defined as an increase of wall-to-lumen ratio, associated with hypertrophy in conductance arteries (Heagerty et al., 1993; Intengan and Schiffrin, 2001), or without medial enlargement in resistance arteries (Mulvany and Halpern, 1977; Gibbons and Dzau, 1994). This type of remodeling, although beneficial in the beginning, may subsequently contribute to cardiovascular disorders. However, chronic alterations in the hemodynamic profile (i.e., chronic changes in blood pressure or blood flow) may potentiate arterial remodeling (Langille, 1996; Lehoux and Tedgui, 1998). In the present study, although DMO rats have an important rise of blood pressure at 18 months of age, we do not detect any modification of internal diameter and MCSA of the thoracic aorta compared to CMO rats. This absence of inward remodeling in case of hypertensive state could be related to the reorganization of the vessel wall (increased connections between VSMCs and ECM), which contributes to the maintenance of a normal level of wall stiffness despite the increase in the pressure wall stress. In a previous study, we also found an absence of inward remodeling of mesenteric resistance arteries in response to this hypertension (Vessières et al., 2016). But we were not able to detect modification in the number of connections between elastic lamellae and ECM in this type of vessel. Nevertheless, in our model of rats exposed *in utero* to maternal diabetes, both resistance (mesenteric) (Vessières et al., 2016) and conductance (thoracic aorta) arteries (present study) are unable to develop inward remodeling in response to high blood pressure.

A chronic decrease in blood flow is also able to induce a reduction in lumen diameter of resistance arteries (inward eutrophic remodeling) (Buus et al., 2001; Baron-Menguy et al., 2010). The model used allows the study of comparable mesenteric resistance arteries submitted to low or normal flow *in vivo* without changes in physiological hemodynamic conditions (i.e., blood pressure). Our results show that ligation of mesenteric resistance arteries, resulting in a low blood flow, does not induce inward remodeling either. Bakker et al. have demonstrated that flow-mediated remodeling is directed by vascular tone (i.e., vasoconstriction inducing inward remodeling in opposition to vasodilatation which induces outward remodeling) (Bakker et al., 2008). Whether it is triggered by high pressure or low blood flow, the resulting inward remodeling requires 3 conditions: first a partial digestion of the ECM, secondly a chronic vasoconstriction and finally a matrix reorganization (Bakker et al., 2002; Langille and Dajnowiec, 2005; Huelsz-Prince et al., 2013). We have previously demonstrated that *in utero* exposure to maternal diabetes resulted in a fetal programming of vascular function in favor of vasoconstrictor tone (Duong Van Huyen et al., 2010; Vessières et al., 2016). Nevertheless, a chronic decrease of blood flow does not induce the development of an inward remodeling in DMO rats. This absence of vascular wall reorganization in response to decreased flow is probably the result of an inability of the vessel to further decrease its diameter. Changes in blood flow induce an inflammatory response responsible for oxidative stress followed by the activation of metalloproteases (MMPs) causing a partial dissociation of the ECM (Vessières et al., 2012). Also, in the case of decreased blood flow, we do not observe a higher increase in ROS production in DMO rats compared to basal conditions or to control animals. This lack of increasing oxidative stress in LF arteries of DMO rats could be responsible for the absence of MMPs activation implicated in a partial dissociation of the ECM and then, the absence of worsening inward remodeling in DMO rats. Furthermore, several studies have shown that vascular remodeling of resistance arteries after reduced blood flow, hypertension, or exposure to vasoconstrictors depends on tissue-TG2 activity to stabilize arterial wall and normalize shear stress (Bakker et al., 2005, 2006; Eftekhari et al., 2007; Pistea et al., 2008). Indeed, the major function of TG2 is to stabilize ECM proteins through the formation of specific cross-links (Bakker et al., 2008). In LF arteries isolated from DMO rats, TG2 activation by DTT is ineffective; by contrast with LF arteries of CMO rats in which DTT increased MMPs activity by 2 times. This absence of TG2 activity is not correlated to a decrease of TG2 protein expression. Although protein cross-linking is the main feature of TG2, providing mechanical strength to tissues (Lorand and Graham, 2003), how vascular smooth muscle tone induces TG2 activity remains unknown (Huelsz-Prince et al., 2013).

## Conclusion

Our study clearly demonstrates that *in utero* exposure to maternal diabetes induces deep architecture vessel wall modifications with matrix reorganization in early stage of life and impacts vascular remodeling mechanisms in the case of chronic hemodynamic changes (i.e., blood pressure or flow). Then, the inability of conductance and resistance arteries to respond to high blood pressure or decreased blood flow could be an adaptive process in order to prevent cardiovascular complications due to programmed hypertension in DMO rats.

## Author contributions

CF: Designed the protocol, obtained grants, researched data, wrote the manuscript, contributed to discussion and edited the manuscript; AD, CP, MM, JB, and LG: Performed the experiments; AD, MM, LL, ZF, and DH: Contributed to discussion and reviewed the manuscript.

### Conflict of interest statement

The authors declare that the research was conducted in the absence of any commercial or financial relationships that could be construed as a potential conflict of interest.

## References

[B1] Abi Khalil C. Travert F. Fetita S. Rouzet F. Porcher R. Riveline J. P.. (2010). Fetal exposure to maternal type 1 diabetes is associated with renal dysfunction at adult age. Diabetes 59, 2631–2636. 10.2337/db10-0419 20622173 PMC3279566

[B2] Aceti A. Santhakumaran S. Logan K. M. Philipps L. H. Prior E. Gale C.. (2012). The diabetic pregnancy and offspring blood pressure in childhood: a systematic review and meta-analysis. Diabetologia 55, 3114–3127. 10.1007/s00125-012-2689-8 22948491

[B3] Alexander B. T. (2006). Fetal programming of hypertension. Am. J. Physiol. Regul. Integr. Comp. Physiol. 290, R1–R10. 10.1152/ajpregu.00417.2005 16352854

[B4] Amri K. Freund N. Vilar J. Merlet-Bénichou C. Lelièvre-Pégorier M. (1999). Adverse effects of hyperglycemia on kidney development in rats: *in vivo* and *in vitro* studies. Diabetes 48, 2240–225. 10.2337/diabetes.48.11.2240 10535460

[B5] Bakker E. N. Buus C. L. Spaan J. A. Perree J. Ganga A. Rolf T. M.. (2005). Small artery remodeling depends on tissue-type transglutaminase. Circ. Res. 96, 119–126. 10.1161/01.RES.0000151333.56089.66 15550691

[B6] Bakker E. N. Buus C. L. VanBavel E. Mulvany M. J. (2004). Activation of resistance arteries with endothelin-1: from vasoconstriction to functional adaptation and remodeling. J. Vasc. Res. 41, 174–182. 10.1159/000077288 15017111

[B7] Bakker E. N. Pistea A. Spaan J. A. Rolf T. de Vries C. J. van Rooijen N.. (2006). Flow-dependent remodeling of small arteries in mice deficient for tissue-type transglutaminase: possible compensation by macrophage-derived factor XIII. Circ. Res. 99, 86–92. 10.1161/01.RES.0000229657.83816.a7 16741156

[B8] Bakker E. N. Pistea A. VanBavel E. (2008). Transglutaminases in vascular biology: relevance for vascular remodeling and atherosclerosis. J. Vasc. Res. 45, 271–28. 10.1159/000113599 18212504

[B9] Bakker E. N. van der Meulen E. T. van den Berg B. M. Everts V. Spaan J. A. VanBavel E. (2002). Inward remodeling follows chronic vasoconstriction in isolated resistance arteries. J. Vasc. Res. 39, 12–20. 10.1159/000048989 11844933

[B10] Baron-Menguy C. Toutain B. Cousin M. Dumont O. Guihot A. L. Vessières E.. (2010). Involvement of angiotensin II in the remodeling induced by a chronic decrease in blood flow in rat mesenteric resistance arteries. Hypertens. Res. 33, 857–866. 10.1038/hr.2010.83 20535114

[B11] Bezie Y. Lacolley P. Laurent S. Gabella G. (1998). Connection of smooth muscle cells to elastic lamellae in aorta of spontaneously hypertensive rats. Hypertension 32, 166–19. 9674655 10.1161/01.hyp.32.1.166

[B12] Blondeau B. Joly B. Perret C. Prince S. Bruneval P. Lelièvre-Pégorier M.. (2011). Exposure in utero to maternal diabetes leads to glucose intolerance and high blood pressure with no major effects on lipid metabolism. Diabetes Metab. 37, 245–51. 10.1016/j.diabet.2010.10.008 21257329

[B13] Bouvet C. Belin de Chantemèle E. Guihot A. L. Vessières E. Bocquet A. Dumont O.. (2007). Flow-induced remodeling in resistance arteries from obese Zucker rats is associated with endothelial dysfunction. Hypertension 50, 248–254. 10.1161/HYPERTENSIONAHA.107.088716 17515452

[B14] Brenner B. M. Garcia D. L. Anderson S. (1988). Glomeruli and blood pressure. Less of one, more the other? Am. J. Hypertens 1(4 Pt. 1), 335–347. 3063284 10.1093/ajh/1.4.335

[B15] Bunt J. C. Tataranni P. A. Salbe A. D. (2005). Intrauterine exposure to diabetes is a determinant of hemoglobin A(1)c and systolic blood pressure in pima Indian children. J. Clin. Endocrinol. Metab. 90, 3225–3229. 10.1210/jc.2005-0007 15797952 PMC1579248

[B16] Buus C. L. Pourageaud F. Fazzi G. E. Janssen G. Mulvany M. J. De Mey J. G. (2001). Smooth muscle cell changes during flow-related remodeling of rat mesenteric resistance arteries. Circ. Res. 89, 180–186. 10.1161/hh1401.093575 11463726

[B17] Cheong J. N. Wlodek M. E. Moritz K. M. Cuffe J. S. (2016). Programming of maternal and offspring disease: impact of growth restriction, fetal sex and transmission across generations. J. Physiol. 594, 4727–4740. 10.1113/JP271745 26970222 PMC5009791

[B18] Cousin M. Custaud M. A. Baron-Menguy C. Toutain B. Dumont O. Guihot A. L.. (2010). Role of angiotensin II in the remodeling induced by a chronic increase in flow in rat mesenteric resistance arteries. Hypertension 55, 109–15. 10.1161/HYPERTENSIONAHA.108.127456 19948989

[B19] Dingemans K. P. Teeling P. Lagendijk J. H. Becker A. E. (2000). Extracellular matrix of the human aortic media: an ultrastructural histochemical and immunohistochemical study of the adult aortic media. Anat Rec 258, 1–14. 10.1002/(SICI)1097-0185(20000101)258:1<1::AID-AR1>3.0.CO;2-7 10603443

[B20] Duong Van Huyen J. P. Vessières E. Perret C. Troise A. Prince S. Guihot A. L.. (2010). In utero exposure to maternal diabetes impairs vascular expression of prostacyclin receptor in rat offspring. Diabetes 59, 2597–2602. 10.2337/db10-0311 20622163 PMC3279527

[B21] Eftekhari A. Rahman A. Schaebel L. H. Chen H. Rasmussen C. V. Aalkjaer C.. (2007). Chronic cystamine treatment inhibits small artery remodelling in rats. J. Vasc. Res. 44, 471–482. 10.1159/000106465 17657163

[B22] El-Mas M. M. Afify E. A. Omar A. G. Mohy El-Din M. M. Sharabi F. M. (2003). Testosterone depletion contributes to cyclosporine-induced chronic impairment of acetylcholine renovascular relaxations. Eur. J. Pharmacol. 468, 217–224. 10.1016/S0014-2999(03)01720-5 12754060

[B23] Gibbons G. H. Dzau V. J. (1994). The emerging concept of vascular remodeling. N. Engl. J. Med. 330, 1431–1438. 8159199 10.1056/NEJM199405193302008

[B24] Gomes G. N. Gil F. Z. (2011). Prenatally programmed hypertension: role of maternal diabetes. Braz. J. Med. Biol. Res. 44, 899–904. 10.1590/S0100-879X2011007500109 21876875

[B25] Heagerty A. M. Aalkjaer C. Bund S. J. Korsgaard N. Mulvany M. J. (1993). Small artery structure in hypertension. Dual processes of remodeling and growth. Hypertension 21, 391–337. 10.1161/01.HYP.21.4.391 8458640

[B26] Hokke S. N. Armitage J. A. Puelles V. G. Short K. M. Jones L. Smyth I. M.. (2013). Altered ureteric branching morphogenesis and nephron endowment in offspring of diabetic and insulin-treated pregnancy. PLoS ONE 8:e58243. 10.1371/journal.pone.0058243 23516451 PMC3596403

[B27] Holemans K. Gerber R. T. Meurrens K. De Clerck F. Poston L. Van Assche F. A. (1999). Streptozotocin diabetes in the pregnant rat induces cardiovascular dysfunction in adult offspring. Diabetologia 42, 81–89. 10.1007/s001250051117 10027583

[B28] Huelsz-Prince G. Belkin A. M. VanBavel E. Bakker E. N. (2013). Activation of extracellular transglutaminase 2 by mechanical force in the arterial wall. J. Vasc. Res. 50, 383–95. 10.1159/000354222 23988702

[B29] Intengan H. D. Schiffrin E. L. (2001). Vascular remodeling in hypertension: roles of apoptosis, inflammation, and fibrosis. Hypertension 38(3 Pt 2), 581–587. 10.1161/hy09t1.096249 11566935

[B30] Jin Y. M. Zhao S. Z. Zhang Z. L. Chen Y. Cheng X. Chuai M.. (2017). High glucose level induces cardiovascular dysplasia during early embryo development. Exp. Clin. Endocrinol. Diabetes. 121, 448–454. 10.1055/s-0033-1349080 23864493

[B31] Langille B. L. (1996). Arterial remodeling: relation to hemodynamics. Can. J. Physiol. Pharmacol. 74, 834–841. 10.1139/y96-082 8946070

[B32] Langille B. L. Dajnowiec D. (2005). Cross-linking vasomotor tone and vascular remodeling: a novel function for tissue transglutaminase? Circ. Res. 96, 9–11. 10.1161/01.RES.0000153883.55971.81 15637303

[B33] Lehoux S. Tedgui A. (1998). Signal transduction of mechanical stresses in the vascular wall. Hypertension 32, 338–45. 9719064 10.1161/01.hyp.32.2.338

[B34] Levy E. Spahis S. Bigras J. L. Delvin E. Borys J. M. (2017). The epigenetic machinery in vascular dysfunction and hypertension. Curr. Hypertens. Rep. 19:52. 10.1007/s11906-017-0745-y 28540644

[B35] Liu N. Q. Ouyang Y. Bulut Y. Lagishetty V. Chan S. Y. Hollis B. W.. (2013). Dietary vitamin D restriction in pregnant female mice is associated with maternal hypertension and altered placental and fetal development. Endocrinology 154, 2270–2280. 10.1210/en.2012-2270 23677931

[B36] Lorand L. Graham R. M. (2003). Transglutaminases: crosslinking enzymes with pleiotropic functions. Nat. Rev. Mol. Cell Biol. 4, 140–56. 10.1038/nrm1014 12563291

[B37] Manderson J. G. Mullan B. Patterson C. C. Hadden D. R. Traub A. I. McCance D. R. (2002). Cardiovascular and metabolic abnormalities in the offspring of diabetic pregnancy. Diabetologia 45, 991–996. 10.1007/s00125-002-0865-y 12136397

[B38] Mulvany M. J. Halpern W. (1977). Contractile properties of small arterial resistance vessels in spontaneously hypertensive and normotensive rats. Circ. Res. 41, 19–26. 10.1161/01.RES.41.1.19 862138

[B39] Nehiri T. Duong Van Huyen J. P. Viltard M. Fassot C. Heudes D. Freund N.. (2008). Exposure to maternal diabetes induces salt-sensitive hypertension and impairs renal function in adult rat offspring. Diabetes 57, 2167–2175. 10.2337/db07-0780 18443204 PMC2494671

[B40] Ojeda N. B. Intapad S. Alexander B. T. (2014). Sex differences in the developmental programming of hypertension. Acta Physiol. 210, 307–316. 10.1111/apha.12206 24268043 PMC4032374

[B41] Pasterkamp G. de Kleijn D. P. Borst C. (2000). Arterial remodeling in atherosclerosis, restenosis and after alteration of blood flow: potential mechanisms and clinical implications. Cardiovasc. Res. 45, 843–852. 10.1016/S0008-6363(99)00377-6 10728409

[B42] Piecewicz S. M. Pandey A. Roy B. Xiang S. H. Zetter B. R. Sengupta S. (2012). Insulin-like growth factors promote vasculogenesis in embryonic stem cells. PLoS ONE 7:e32191. 10.1371/journal.pone.0032191 22363814 PMC3283730

[B43] Pistea A. Bakker E. N. Spaan J. A. Hardeman M. R. van Rooijen N. VanBavel E. (2008). Small artery remodeling and erythrocyte deformability in L-NAME-induced hypertension: role of transglutaminases. J. Vasc. Res. 45, 10–8. 10.1159/000109073 17898543

[B44] Porto N. P. Jucá D. M. Lahlou S. Coelho-de-Souza A. N. Duarte G. P. Magalhães P. J. (2010). Effects of K^+^channels inhibitors on the cholinergic relaxation of the isolated aorta of adult offspring rats exposed to maternal diabetes. Exp. Clin. Endocrinol. Diabetes 118, 360–363. 10.1055/s-0029-1241824 20397125

[B45] Ramos-Alves F. E. de Queiroz D. B. Santos-Rocha J. Duarte G. P. Xavier F. E. (2012). Effect of age and COX-2-derived prostanoids on the progression of adult vascular dysfunction in the offspring of diabetic rats. Br. J. Pharmacol. 166, 2198–2208. 10.1111/j.1476-5381.2012.01945.x 22436072 PMC3402782

[B46] Retailleau K. Toutain B. Galmiche G. Fassot C. Sharif-Naeini R. Kauffenstein G.. (2013). Selective involvement of serum response factor in pressure-induced myogenic tone in resistance arteries. Arterioscler. Thromb. Vasc. Biol. 33, 339–346. 10.1161/ATVBAHA.112.300708 23264443

[B47] Rocha S. O. Gomes G. N. Forti A. L. do Carmo Pinho Franco M. Fortes Z. B. de Fátima Cavanal M.. (2005). Long-term effects of maternal diabetes on vascular reactivity and renal function in rat male offspring. Pediatr. Res. 58, 1274–129. 10.1203/01.pdr.0000188698.58021.ff 16306207

[B48] Romano T. Wark J. D. Wlodek M. E. (2015). Developmental programming of bone deficits in growth-restricted offspring. Reprod. Fertil. Dev. 27, 823–833. 10.1071/RD13388 24613152

[B49] Sack M. N. Fyhrquist F. Y. Saijonmaa O. J. Fuster V. Kovacic J. C. (2017). Basic biology of oxidative stress and the cardiovascular system: part 1 of a 3-part series. J. Am. Coll. Cardiol. 70, 196–211. 10.1016/j.jacc.2017.05.034 28683968 PMC5551687

[B50] Sauder K. A. Hockett C. W. Ringham B. M. Glueck D. H. Dabelea D. (2017). Fetal overnutrition and offspring insulin resistance and beta-cell function: the Exploring Perinatal Outcomes among Children (EPOCH) study. Diabet. Med. 34, 1392–139. 10.1111/dme.13417 28636758 PMC5603388

[B51] Simeoni U. Barker D. J. (2009). Offspring of diabetic pregnancy: long-term outcomes. Semin. Fetal Neonatal Med. 14, 119–124. 10.1016/j.siny.2009.01.002 19208505

[B52] Szostak-Wegierek D. (2014). Intrauterine nutrition: long-term consequences for vascular health. Int. J. Womens. Health 6, 647–656. 10.2147/IJWH.S48751 25050077 PMC4103922

[B53] Tain Y. L. Lin Y. J. Sheen J. M. Yu H. R. Tiao M. M. Chen C. C.. (2017). High fat diets sex-specifically affect the renal transcriptome and program obesity, kidney injury, and hypertension in the offspring. Nutrients 9:357. 10.3390/nu9040357 28368364 PMC5409696

[B54] Vessières E. Dib A. Bourreau J. Lelièvre E. Custaud M. A. Lelièvre-Pégorier M.. (2016). Long lasting microvascular tone alteration in rat offspring exposed in utero to maternal hyperglycaemia. PLoS ONE 11:e0146830. 10.1371/journal.pone.0146830 26756337 PMC4710502

[B55] Vessières E. Freidja M. L. Loufrani L. Fassot C. Henrion D. (2012). Flow (shear stress)-mediated remodeling of resistance arteries in diabetes. Vascul. Pharmacol. 57, 173–178. 10.1016/j.vph.2012.03.006 22484164

[B56] Weiss C. Passow H. Rothstein A. (1959). Autoregulation of flow in isolated rat kidney in the absence of red cells. Am. J. Physiol. 196, 1115–1118. 13649942 10.1152/ajplegacy.1959.196.5.1115

[B57] Wichi R. B. Souza S. B. Casarini D. E. Morris M. Barreto-Chaves M. L. Irigoyen M. C. (2005). Increased blood pressure in the offspring of diabetic mothers. Am. J. Physiol. Regul. Integr. Comp. Physiol. 288, R1129–R133. 10.1152/ajpregu.00366.2004 15661971

