# Supplementary material for: In Utero Exposure to Maternal Diabetes Is Associated With Early Abnormal Vascular Structure in Offspring
Source: Front Physiol. 2018 Apr 4;9:350. doi: 10.3389/fphys.2018.00350 (PMC5893798; doi:10.3389/fphys.2018.00350)

Figure S1: *Evolution of plasma glucose concentration in fasted pregnant female rats after streptozotocin injection (diabetic mother, n=18) or citrate buffer injection (control mother, n=14) every 2 days before delivery.* *** $p<0.001$ diabetic mother vs control mother.

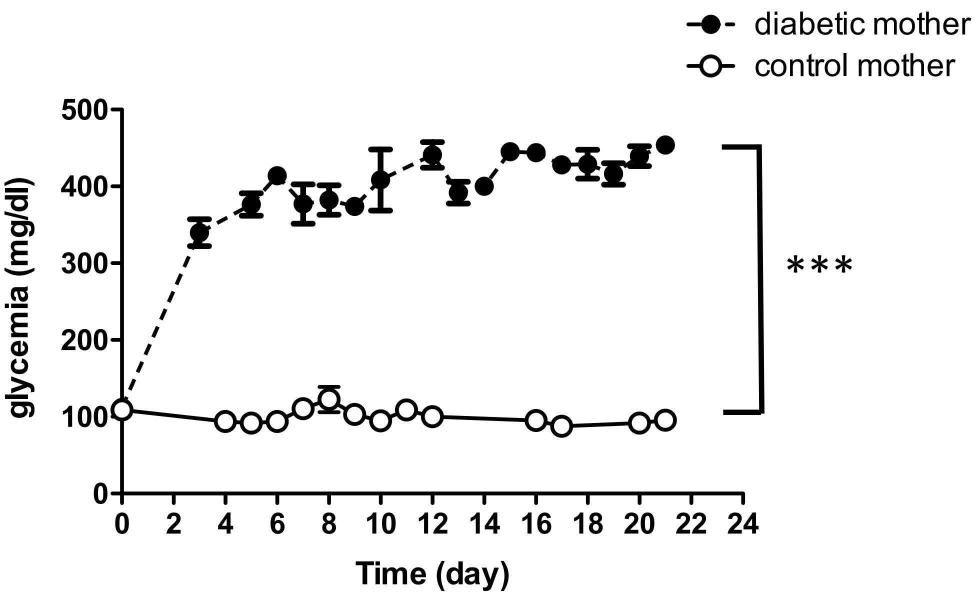

A

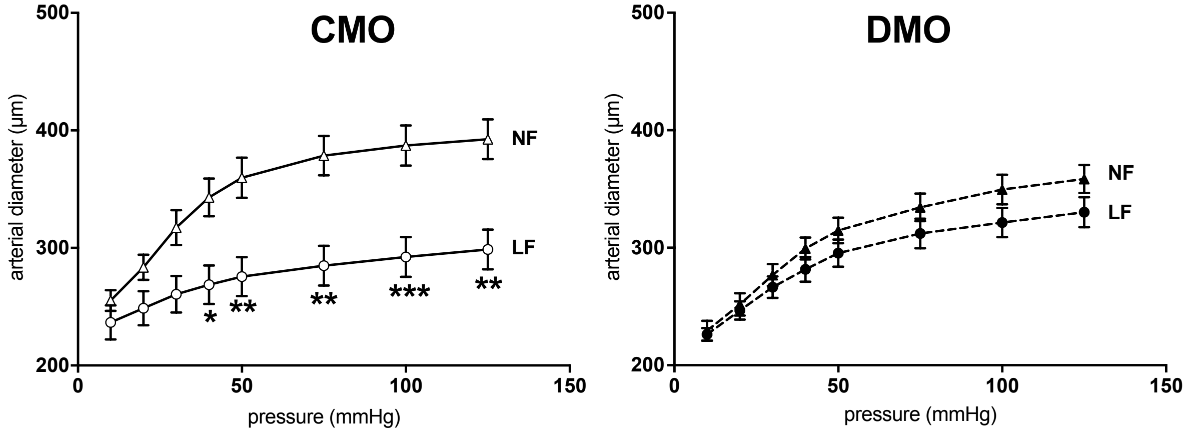

B

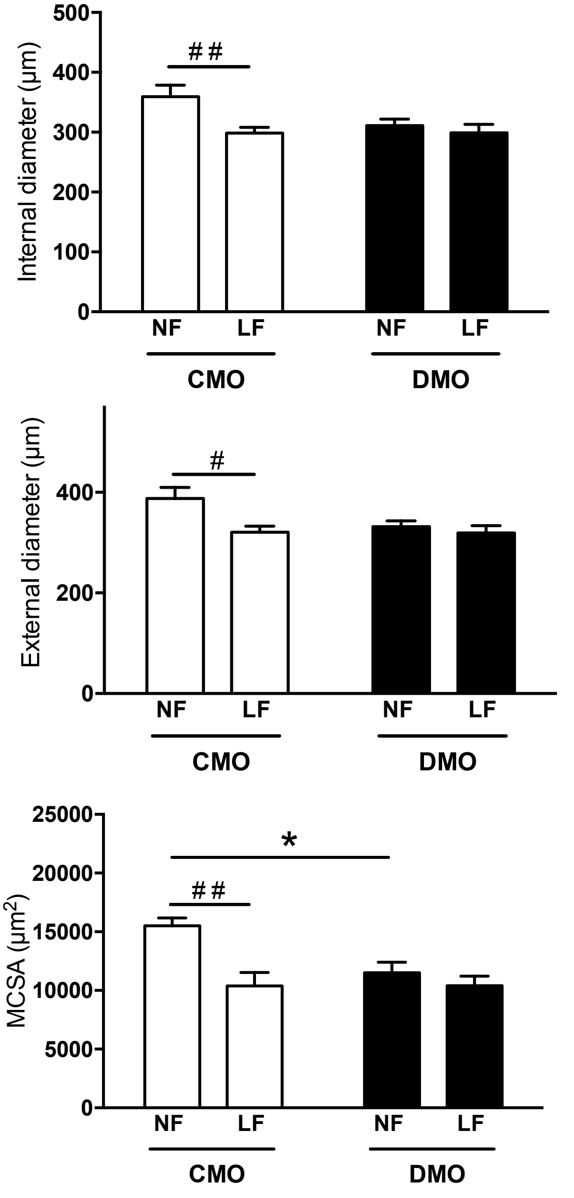

Figure S2: *Flow remodeling analysis of mesenteric arteries in 3-month old control (CMO) and diabetic (DMO) mother offspring* ***21 days*** *after ligation.* (A) Low flow (LF) and normal flow (NF) arteries diameters of CMO and DMO in response to stepwise increase pressure (n=7), * p<0.05, **p<0.01 and *** p<0.001 LF vs NF. (B) Morphometric measurements of internal and external diameters and cross-sectional area of the media (MCSA) of NF and LF arteries in CMO (open bars, n=8) and DMO (solid bars, n=7). Each bar graph represents mean ± SEM. * p<0.05 DMO vs CMO, # p<0.05, ## p<0.01 LF vs NF.

Figure S3: *Protein expression analysis in normal flow (NF) and low flow (LF) mesenteric arteries of 3-month old control (CMO, n=6) and diabetic (DMO, n=6) mother offspring.* (A and C) Nitrocellulose membrane stained with Ponceau S dye for protein detection with a molecular weight marker (PM) on the left and (B and D) Blotting gels of SOD2, transglutaminase 2 (TG2, bottom line of the blot indicating by an narrow) and GAPDH (control protein).

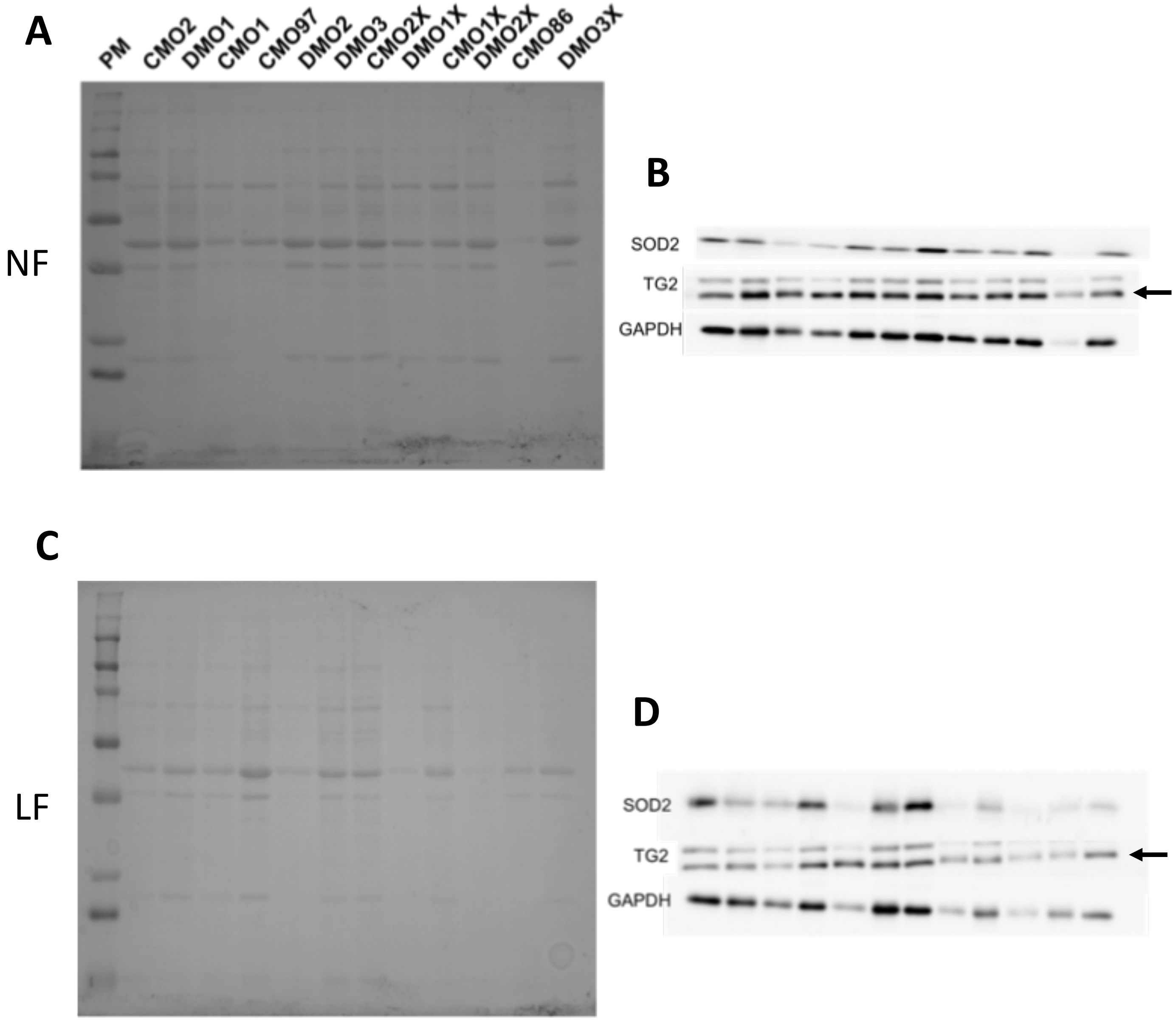

Supplement: Supplementary file 1 [file Image1.pdf]
